# Airway Bacteria Quantification Using Polymerase Chain Reaction Combined with Neutrophil and Eosinophil Counts Identifies Distinct COPD Endotypes

**DOI:** 10.3390/biomedicines9101337

**Published:** 2021-09-27

**Authors:** Augusta Beech, Simon Lea, Jian Li, Natalie Jackson, Alex Mulvanny, Dave Singh

**Affiliations:** 1 Manchester Academic Health Science Centre, Division of Infection, Immunity and Respiratory Medicine, School of Biological Sciences, Faculty of Biology, Medicine and Health, The University of Manchester, Manchester M13 9PL, UK; simon.lea@manchester.ac.uk (S.L.); Jian.Li@manchester.ac.uk (J.L.); amulvanny@meu.org.uk (A.M.); dsingh@meu.org.uk (D.S.); 2 Medicines Evaluation Unit, Manchester University NHS Foundation Trust, Manchester M23 9QZ, UK; njackson@meu.org.uk

**Keywords:** chronic obstructive pulmonary disease, inflammatory endotypes, *Haemophilus influenzae*, eosinophil, eosinophilic inflammation, neutrophil, neutrophilic inflammation, sputum, airway colonisation

## Abstract

Background: Chronic obstructive pulmonary disease (COPD) inflammatory endotypes are associated with different airway microbiomes. We used quantitative polymerase chain reaction (qPCR) analysis of sputum samples to establish the bacterial load upper limit in healthy controls; these values determined the bacterial colonisation prevalence in a longitudinal COPD cohort. Bacteriology combined with sputum inflammatory cells counts were used to investigate COPD endotypes. Methods: Sixty COPD patients and 15 healthy non-smoking controls were recruited. Sputum was analysed by qPCR (for *Haemophilus influenzae, Moraxella catarrhalis, Streptococcus pneumoniae* and *Psuedomonas aeruginosa*) and sputum differential cell counts at baseline and 6 months. Results: At baseline and 6 months, 23.1% and 25.6% of COPD patients were colonised with *H. influenzae*, while colonisation with other bacterial species was less common, e.g., *S. pneumoniae*—1.9% and 5.1%, respectively. *H. influenzae* + ve patients had higher neutrophil counts at baseline (90.1% vs. 67.3%, *p* < 0.01), with similar results at 6 months. COPD patients with sputum eosinophil counts ≥3% at ≥1 visit rarely showed bacterial colonisation. Conclusions: The prevalence of *H. influenzae* colonisation was approximately 25%, with low colonisation for other bacterial species. *H. influenzae* colonisation was associated with sputum neutrophilia, while eosinophilic inflammation and *H. influenzae* colonisation rarely coexisted.

## 1. Introduction

Chronic obstructive pulmonary disease (COPD) is characterised by heterogeneous airway inflammation [1,2,3]. Neutrophilic airway inflammation is a common feature in COPD patients, but the magnitude of neutrophil influx into the lungs varies between individuals [4,5]. Furthermore, eosinophilic airway inflammation is present in a subset of COPD patients, and is associated with a profile of T2 inflammation and increased responsiveness to inhaled corticosteroid (ICS) treatment [1,6,7]. A disease endotype is a subgroup of individuals who display a distinct biological mechanism [8], such as eosinophilic inflammation in COPD [1,9]. Identifying endotypes can help the targeting of pharmacological treatments towards patients who are most likely to gain therapeutic benefit (“precision medicine”) [10].

Microbiome studies using 16S rRNA sequencing have demonstrated dysbiosis in sputum and bronchial brush samples of COPD patients compared to healthy controls [11,12,13]. Increased abundance of the Proteobacteria and Firmicutes phyla in COPD patients have been reported, with increased *Haemophilus*, *Moraxella* and *Streptococcus* identified at the genera level [13]. These findings are consistent with clinical observations using bacterial culture that COPD patients have increased susceptibility to infection with *Haemophilus influenzae* (*H. influenzae*), *Moraxella catarrhalis* (*M. catarrhalis*) and *Streptococcus pneumoniae* (*S. pneumoniae*) [14,15].

Quantitative polymerase chain reaction (qPCR) and 16S rRNA sequencing of COPD sputum samples obtained in the stable state have shown that the presence of *Haemophilus* is associated with increased neutrophilic airway inflammation; this association was not observed for other bacteria [3,16,17]. *Haemophilus* presence in the stable state is also associated with lower sputum eosinophil counts [3,18]. *Haemophilus*, therefore, appears to skew the airway immune response towards a neutrophil dominant endotype and away from an eosinophilic endotype.

Sputum qPCR analysis has been advocated as a more sensitive method for bacterial identification and quantitation compared to bacterial culture [16,19]. qPCR analysis also allows absolute quantification of bacterial presence, in contrast to 16S rRNA sequencing, which provides information on relative abundance within a sample. However, the qPCR thresholds used to define clinically significant bacterial presence in the stable state, in other words colonisation, have varied between studies [3,16,17,20] and have not been defined based on a healthy control range. It is logical that colonisation in the COPD stable state using qPCR analysis should be defined as levels greater than those found in the normal healthy microbiome; previous studies using other thresholds may have over- or under-estimated the proportion of COPD patients with bacterial colonisation. 

In this longitudinal COPD cohort study, we used a healthy control group to define a normal range for bacterial species quantification. This novel approach allowed more precise identification of COPD patients with increased bacterial presence in the airways. We investigated the prevalence of COPD endotypes based on combined analysis of sputum inflammatory cell counts and bacterial species identification by qPCR, with a particular focus on identifying the prevalence of the *H. influenzae*/neutrophil-dominant endotype and whether the eosinophilic endotype occurs independently of *H. influenzae* colonisation.

## 2. Materials and Methods

### 2.1. Study Cohort

COPD patients and healthy non-smoking (HNS) controls were recruited from the Medicines Evaluation Unit (Manchester University NHS Foundation Trust). All subjects (COPD patients and HNS) were aged ≥40 years old. None of the participants were using maintenance antibiotics or oral corticosteroids and they had no previous asthma diagnosis. COPD patients had a smoking history of ≥10 pack years and were included if highly symptomatic with a Modified Medical Research Council questionnaire (mMRC) [21] score ≥2 and COPD assessment test (CAT) [22] score >15. HNS had a ratio of forced expiratory volume in one second to forced vital capacity (FEV_1_/FVC ratio) of >0.7, no history of respiratory disease and were non-smokers with a pack year history of <1. All patients provided written informed consent using protocols approved by local Ethics Committees (16/NW/0836, 05/Q1402/41 and 10/H1016/25).

### 2.2. Study Design 

Sputum samples were obtained from participants during stable state, described as no symptom-defined exacerbation or respiratory illness within 4 weeks of sampling. Symptoms were assessed using CAT and mMRC scores and health related quality of life using the St George’s Respiratory Questionnaire (SGRQ-C) [23]. Lung function measurements were performed according to guidelines [24,25]. 

### 2.3. Sputum Measurements 

Sputum induction was performed, and spontaneous samples were collected, where FEV_1_ was <800 mL (approximately 3% of samples). Sputum was processed for real-time qPCR detection of absolute abundance for the following bacterial species: *H. influenzae, M. catarrhalis*, *S. pneumoniae* and *Pseudomonas aeruginosa* (*P. aeruginosa*), as previously described [26]. Briefly, selected sputum plugs were processed preferentially by homogenisation with phosphate-buffered saline (PBS) and glass beads. The remaining sample was processed following a two-step method using Dulbecco’s phosphate-buffered saline (D-PBS), then a dithiothreitol (DTT) step allowing for preparation of cytospins for differential cell counts (DCC) as previously described [27]. Small sputum samples (minimum weight of approximately 0.1 g) were preferentially processed for bacterial qPCR analysis only. Details are in the Supplemental Materials.

### 2.4. qPCR Detection of Common Respiratory Pathogens

DNA was extracted from homogenised sputum samples using QIAamp DNA mini Kit (QIAGEN, Crawley, West Sussex, UK) [28]; bacterial DNA was stored at −80 °C. Real-time qPCR was performed on *H. influenzae, M. catarrhalis, S. pneumoniae* and *P. aeruginosa*, targeting the lipo-oligosaccharide glycosyltransferase-encoding gene (lgtC) of *H. influenzae*, the CopB outer membrane protein-encoding gene of *M. catarrhalis*, the autolysin-encoding gene (lytA) of *S. pneumoniae* and the gyrB gene of *P. aeruginosa* as previously described [26,28]. Details are in the Supplemental Materials, including details of primers and probes (Table S1). 

### 2.5. Statistical Analysis

The upper limits of HNS colonisation were used as a threshold to define bacterial colonisation for individual bacterial species in COPD patients. Parametric and nonparametric data are presented as mean (standard deviation, SD) and median [range], respectively. All qPCR data for bacterial loads were non-parametric; comparisons were performed using the Mann–Whitney U test. Clinical characteristics were analysed using a Student’s *t*-test or a Mann–Whitney U test depending on normality of the data. Categorical analyses of clinical characteristics and comparisons across groups were performed using a Chi-squared test. *p* < 0.05 was considered statistically significant. Analyses were performed using version 9.00, GraphPad Prism (San Diego, CA, USA).

## 3. Results

Sixty COPD patients and 15 healthy controls were recruited. In total, 52 COPD and 15 healthy controls provided a sputum sample at baseline. From 39 COPD patients, 6-month follow-up samples were obtained, of whom 31 had matched samples from both visits. There were 14 COPD patients who withdrew from longitudinal follow-up for personal reasons or deterioration in health (Figure S1), thus reducing the number of matched samples available. The baseline demography and sputum data for both groups are presented in Table 1. COPD patients were older than HNS (mean ages 64.9 vs. 59.0 years, respectively, *p* = 0.02), and no associations between age and *H. influenzae* or total bacterial load were observed in COPD and HNS (Figure S2). COPD patients had a significantly higher number of patients with concomitant diseases including ischemic heart disease, hypertension, hypercholesterolemia and osteoarthritis (Table S2). In the COPD group, the mean FEV_1_ was 66.7% predicted, while the mean SGRQ and median CAT scores were 54.2 and 22.3, respectively. The mean retrospective exacerbation rate for the 12 months prior to recruitment was 1.1 with a prospective annualised rate of 1.2. The proportion of COPD patients using ICS was 71.7%, with no differences in clinical characteristics, sputum cell counts or bacteriology between ICS users and non-users (Table S3), while bacteriology was not different between COPD current and ex-smokers (Table S4). 

The sputum DCC were similar between COPD patients and HNS, including neutrophil counts (69.1% and 70.5%, respectively, *p* = 0.32), apart from higher sputum eosinophil percentage and total eosinophil count/gram in COPD patients (Table 1, *p* < 0.01 for both). 

### 3.1. Bacterial Colonisation 

**Figure 1:**
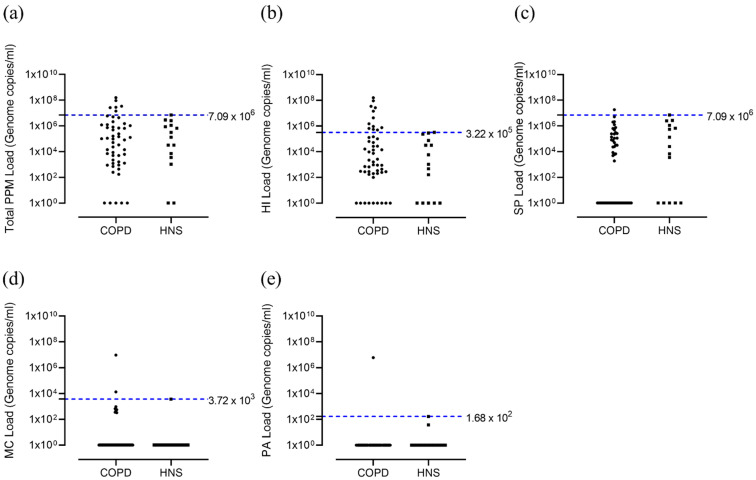
Total bacterial load and load for different PPMs; COPD vs. HNS controls at baseline. Sputum from COPD patients (*n* = 52) at baseline visit and healthy non-smokers (*n* = 15) were analysed for qPCR for presence of *H. influenzae*, *S. pneumoniae*, *M. catarrhalis* and *P. aeruginosa.* Data represent bacterial genome copies for individual patients for (**a**) total bacterial load and that for different bacterial species; (**b**) *H. influenzae*, (**c**) *S. pneumoniae*, (**d**) *M. catarrhalis* and (**e**) *P. aeruginosa*. Chronic obstructive pulmonary disease, COPD; healthy non-smoker, HNS; *Haemophilus influenzae*, HI; *Moraxella catarrhalis*, MC; *Pseudomonas aeruginosa*, PA; potentially pathogenic microorganism, PPM; *Streptococcus pneumoniae*, SP.

Bacterial quantification revealed no significant differences between COPD patients and HNS in the levels of *H. influenzae*, *M. catarrhalis*, *S. pneumoniae* or *P. aeruginosa* (*p* > 0.05 for all comparisons, Table 1). Using the upper threshold of the HNS range, we identified a subgroup of COPD patients (12 out of 52; 23.1%) with *H. influenzae* levels above the HNS range (Figure 1b). There were far fewer COPD patients above the HNS range for *S. pneumoniae* (*n* = 1, 1.9%), *M. catarrhalis* (*n* = 2, 3.8%) and *P. aeruginosa* (*n* = 2, 3.8%) (Figure 1c–e, respectively). We defined *H. influenzae*-positive (HI^+ve^) and -negative (HI^−ve^) COPD patients above and below the upper threshold of the HNS range (3.22 × 10^5^ genome copies/mL), respectively; 2 patients in the HI^+ve^ group were also colonised with either *M. catarrhalis* or both *M. catarrhalis* + *S. pneumoniae*. The clinical characteristics of the HI^+ve^ and HI^−ve^ groups at baseline were mostly similar (detail in the online supplement; Table S5). 

**Figure 2:**
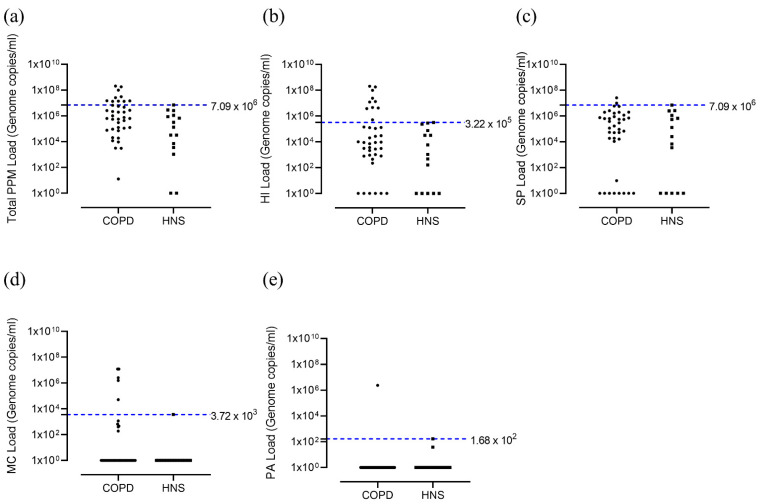
Total bacterial load and load for different PPMs; COPD vs. HNS controls at 6 months. Sputum from COPD patients (*n* = 39) at 6 months visit and healthy non-smokers (*n* = 15) were analysed for qPCR for presence of *H. influenzae*, *S. pneumoniae*, *M. catarrhalis* and *P. aeruginosa.* Data represent bacterial genome copies for individual patients for (**a**) total bacterial load and that for different bacterial species; (**b**) *H. influenzae*, (**c**) *S. pneumoniae*, (**d**) *M. catarrhalis* and (**e**) *P. aeruginosa*. COPD, Chronic obstructive pulmonary disease; HNS, healthy non-smoker; HI, *Haemophilus influenzae*; MC, *Moraxella catarrhalis*; PPM, potentially pathogenic microorganism; PA, *Pseudomonas aeruginosa*; SP, *Streptococcus pneumoniae*.

At 6 months, 10 out of 39 patients were HI^+ve^ (25.6%, Figure 2b), while 2 (5.1%) were colonised with *S. pneumoniae*, 5 (12.8%) with *M. catarrhalis* and 1 (2.6%) with *P. aeruginosa* (Figure 2c–e, respectively).

### 3.2. Relationship between Colonisation and Sputum Cell Counts

**Figure 3:**
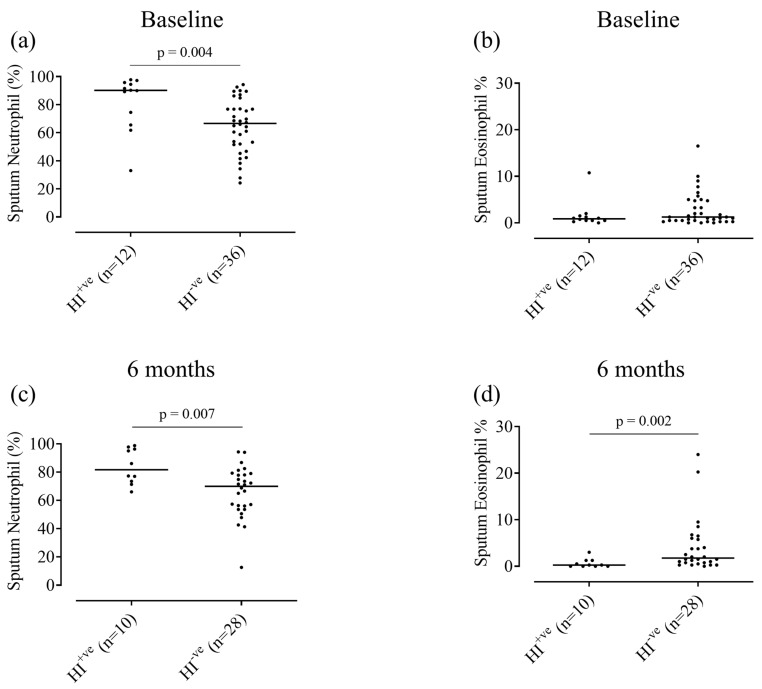
Sputum neutrophil and eosinophil percentages for *Haemophilus influenzae*-positive vs. -negative groups, at baseline and 6 months. Sputum differential cell counts were performed on *H. influenzae*-positive (HI^+ve^) and *H. influenzae*-negative (HI^−ve^) patients at baseline (*n* = 12 and 36, respectively) and 6 months (*n* = 10 and 28, respectively). Data represent sputum neutrophil or eosinophil percentages for individual patients (**a**,**b**) at baseline or (**c**,**d**) at 6 months. Solid black line represents median values. HI, *Haemophilus influenzae*.

At baseline, HI^+ve^ patients had a higher neutrophil percentage and cell count/g compared to HI^−ve^ patients; 90.1% and 67.3%, respectively, *p* < 0.01, and 14.7 × 10^6^/g and 4.7 × 10^6^/g, respectively, *p* = 0.02 (Figure 3a and Table S5). Sputum eosinophils showed a numerical difference between HI^+ve^ and HI^−ve^ patients (0.9 and 1.3%, respectively, Figure 3b) which was not statistically significant (*p* = 0.44). At 6 months, similar results were observed (Figure 3c,d and Table S6); sputum neutrophil percentage and cell count/g were higher in HI^+ve^ patients compared to HI^−ve^ (81.6 vs. 69.9% and 8.5 vs. 4.7 × 10^6^/g, *p* < 0.01 and 0.01, respectively) and sputum eosinophil % was lower in HI^+ve^ patients compared to HI^−ve^ (0.3 vs. 1.8%, respectively, *p* < 0.01), although no significant differences in absolute eosinophil counts were observed. Further details of differences in inflammatory cell counts and bacterial loads are provided in the Supplementary Material. 

**Figure 4:**
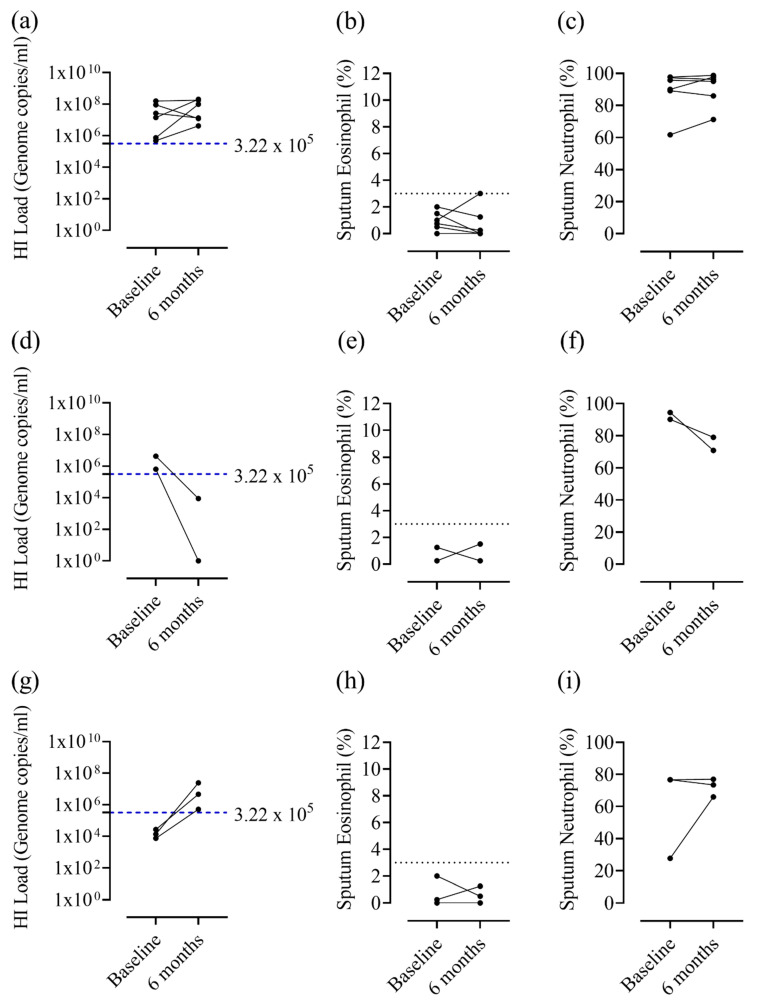
*Haemophilus influenzae* load, sputum neutrophil percentage and eosinophil percentage at baseline and 6-month visits for patients positive for *H. influenzae* at one or both visits. Sputum qPCR and differential cell counts were performed to determine *H. influenzae* load, sputum neutrophil % and eosinophil % at baseline and 6 month visits for patients positive for *H. influenzae* at (**a**–**c**) both visits, (**d**–**f**) baseline only or (**g**–**i**) 6 months only. The blue dashed line represents the upper threshold of the HNS range for *H. Influenzae* (3.22 × 10^5^ genome copies/mL), while the black dotted line indicates 3% sputum eosinophils. HI, *Haemophilus influenzae*.

When data were visually inspected, patients who remained HI^+ve^ from baseline to 6 months had relatively stable sputum neutrophil and eosinophil counts (eosinophil counts remaining mostly below 3%, Figure 4a,c). In contrast, COPD patients who changed *H. influenzae* colonisation status displayed more variable neutrophil counts (Figure 4d,i). No formal statistical analyses were performed due to small subgroup sample sizes. 

COPD patients with a sputum eosinophil count of ≥3% at one or both visits over 6 months rarely showed colonisation with any of the bacterial species studied (using the HNS range to define colonisation), with non-eosinophilic patients (sputum eosinophil count of <3% at both visits) showing significantly greater overall bacterial colonisation at baseline and 6 months (Table 2, *p* = 0.03 for both). COPD patients with a sputum eosinophil count ≥3% at one or both visits were rarely colonised with *H. influenzae* (1 out of 13 samples at baseline and 1 out of 15 samples at 6 months), with a reduced probability of *H. influenzae* colonisation compared to non-eosinophilic patients (8 out of 21 samples at baseline and 8 out of 22 samples at 6 months, *p* = 0.05 and 0.06, respectively). 

## 4. Discussion

This study used a healthy control group to define the upper limit of ‘normal’ bacterial colonisation. Applying this threshold to COPD patients revealed that *H. influenzae* colonisation was present in approximately 25% of individuals, with a similar proportion observed at baseline and 6 month follow-up. In contrast, colonisation with *S. pneumoniae, M. cattarrhalis* and *P. aeruginosa* were less frequent. In agreement with previous studies, *H. influenzae* colonisation was associated with greater neutrophilic airway inflammation and less eosinophilic inflammation [2,3]. Notably, COPD patients with eosinophilic inflammation rarely displayed *H. influenzae* colonisation, providing further evidence that eosinophilic COPD patients have a distinct microbiome [2,3].

### 4.1. Prevalence of Bacterial Colonisation

Using a healthy control group to define colonisation thresholds resulted in bacterial species-specific threshold levels being applied to the COPD cohort. This contrasts with previous studies where a ‘one size fits all’ approach has been used for thresholds, commonly 1 × 10^4^ and 1 × 10^6^ genome copies/mL [3,16,17,26]. These thresholds originate from either (1) the minimum load at which bacteria can be detected using qPCR or (2) the threshold of detection, which shares a high concordance with positive routine culture methods [16,26]. Our results indicate that a more refined approach is appropriate, using different thresholds for each bacterial species.

The prevalence of *H. influenzae* colonisation within our COPD cohort was approximately 23 and 25% at baseline and 6 months, respectively, using the threshold of 3.22 × 10^5^ genome copies/mL. Previous studies have reported a prevalence between 17.4–34% using thresholds of 1 × 10^4^ and 1 × 10^6^ genome copies/mL [3,16,17,20,26]. The threshold defined in the current study lies within the range of 1 × 10^4^ and 1 × 10^6^ genome copies/mL, and consequently, the proportion of patients with colonisation (approximately 25%) lies within the range previously reported (17–34%).

We observed a prevalence of <2% for *S. pneumoniae* colonisation, which is lower than previously reported in COPD studies using PCR (3–33.3% using thresholds of 1 × 10^4^ and 1 × 10^6^) [3,16,17,20,26]. The differences can be attributed to the higher threshold defined in this study (7.09 × 10^6^ genome copies/mL). Respiratory microbiome studies in healthy individuals have reported Streptococcus as one of the most abundant genera [29,30]. It is, therefore, not surprising that we observed a higher level of *S. pneumoniae* presence (relative to *H. influenzae* and *M. catarrhalis*) in our healthy control group. These observations underscore the importance of using bacterial species-specific thresholds for the purpose of defining bacterial loads that are higher than those observed in healthy subjects. 

*M. catarrhalis* detection was low (approximately 3–5%), and similar to the results we published recently in a different cohort (1.7%) [3]. Higher prevalence has been reported in other studies, e.g., 7.4–16% using thresholds of 1 × 10^4^ and 1 × 10^6^ [16,17,19,27]. The threshold defined in the current study (3.72 × 10^3^ genome copies/mL) was lower than these other studies, and thus, cannot account for the lower prevalence reported here. Differences between studies are more likely to be associated with clinical features, including higher exacerbation rates in some other cohorts [15,31]. *P. aeruginosa* detection was minimal in this cohort, in agreement with other studies [16,20,26].

qPCR has been advocated as a more sensitive measure of bacterial identification compared to bacterial culture [19,20,32]. However, without using appropriate thresholds, this method may be over-sensitive. The results presented here provide an opportunity to refine the use of qPCR in the analysis of COPD microbiology to optimise assay sensitivity. It should be noted that several recent COPD cohort studies have utilised 16SrRNA sequencing to describe the respiratory microbiome [2,3,13,18]; these studies provide valuable information on the relative abundance of different bacterial phyla and genera. qPCR enables more accurate quantification at the species level.

### 4.2. H. influenzae and Airway Inflammation 

Consistent with previous findings, patients colonised with *H. influenzae* had evidence of more neutrophilic airway inflammation [2,3,16]. *H. influenzae* colonisation has been associated with elevated sputum pro-inflammatory markers in COPD such as CXCL8, IL-1β, TNF-α and MPO [2,20]. Furthermore, activation of the IL-6 trans-signalling pathway has been associated with the haemophilus genera [33]. Overall, these results indicate that *H. influenzae* may elicit the production of a distinct inflammatory milieu within the lung, which promotes excessive neutrophilic inflammation.

It has been shown that temporal changes in microbiome, measured by 16S rRNA sequencing, show concordant changes in airway inflammation parameters [2]. We have shown previously that *H. influenzae* load and neutrophil % show concordance when observed over time [3]. Here, we report similar observations with regards to change in *H. influenzae* colonisation status over time and neutrophil percentages; while the small sample size prevented statistical analysis, it appeared that a change in *H. influenzae* colonisation status over time could result in a change in neutrophil counts, while stable (high) neutrophilic inflammation was observed in patients with persistent *H. influenzae* colonisation. 

### 4.3. Eosinophilic Airway Inflammation and Bacterial Colonisation

Studies have shown that *H. influenzae* colonisation is associated with low eosinophil counts [2,3]. Here, we show similar results; COPD patients with higher eosinophil counts rarely showed evidence of *H. influenzae* colonisation. Wang et al. reported that patients with an eosinophilic endotype rarely transition to a *H. influenzae*/neutrophilic-dominant endotype, suggesting the two are mutually exclusive [2]. Our results are in agreement, demonstrating that COPD patients with eosinophilic inflammation are generally distinct from those with *H. influenzae* colonisation. 

Previous studies have reported an association between T2 inflammation and higher blood eosinophil counts in COPD [7,34]. We have recently shown that eosinophil^HIGH^ COPD, defined using both blood and sputum eosinophil counts, is associated with increased expression of the T2 genes CLCA1, CCL26, IL−13 and CST1 [6]. The clinical benefits of ICS are likely to be mediated, at least partly, by the suppression of eosinophil-associated T2 inflammation [1], providing an explanation for the association between ICS effects and blood eosinophil counts. 

Eosinophil^HIGH^ COPD patients have higher levels of airway immunoglobulins; IgA, secretory IgA, IgM and IgG1, which is associated with an increased ability to opsonise *H. influenzae* compared to eosinophil^LOW^ COPD patients [35]. These findings provide a potential mechanistic explanation for differences in *H. influenzae* colonisation between eosinophilic and non-eosinophilic COPD described here, based on sputum eosinophil counts > 3%. It should also be considered that CCL26 and CST1 may exert a protective effect against *H. influenzae* via antibacterial activity and protection of tight junction integrity, respectively [36,37]. 

### 4.4. Limitations

The HNS control group were well matched to COPD in terms of BMI and gender, but were slightly younger than the COPD group (59.0 vs. 64.9 years, respectively). The lung microbiome may change with ageing [38,39], and it is possible that some differences in bacterial load reported here between COPD patients and HNS could be attributed to age, although we doubt that the small difference in years could account for our findings, and we found no association between age and bacterial colonisation. Sputum neutrophil counts are known to increase with age (particularly between the ages of 50–59 years) [40], and HNS sputum neutrophil counts were numerically higher than COPD patients; however, the older age of our HNS cohort provides an explanation for this. 

The healthy control group in this study were non-smokers. Smoking in healthy individuals is associated with a trend towards differences in the lung microbiome, although results are conflicting [11,13,41,42]. A healthy smoking control group in the current study may have added some value in distinguishing differences due to COPD vs. those due to active smoking.

We found no microbiome differences associated with ICS use in COPD patients within this cohort. Other studies have reported the same observation [3,16,17,26], although there are conflicting studies showing increased bacterial loads and dysbiosis associated with ICS use [12,26,43,44]. Differences between studies may relate to sample sizes, patient characteristics and analytical methods.

The sample sizes for this study were limited, with some sub-groups being too small for statistical analysis. This was further affected by the inability of some participants to produce sufficient sputum samples during longitudinal data collection. With 15 HNS, we chose a conservative approach to define normal bacterial levels by using the range. We considered our sample size too small to use two standard deviations; larger studies in HNS would be helpful to confirm our thresholds. Despite the limited sample size, the current study benefits from being a single-centre study, thus limiting the variability that may occur between sites. Furthermore, the study design included longitudinal analysis, which allowed for analysis of the repeatability of observations. 

The study population here had a high burden of symptoms, due to the inclusion criteria. It would be important for future studies to determine if the current data are generalisable to broader COPD populations.

## 5. Conclusions

We used healthy controls to determine bacterial species-specific qPCR thresholds for analysing bacterial colonisation in COPD patients. Our main findings were that (1) a subgroup of COPD patients (approximately 25%) display *H. influenzae* colonisation and increased neutrophilic inflammation, while colonisation with other bacterial species was less common, (2) this *H. influenzae*/neutrophilic endotype was stable in some individuals over 6 months of follow-up and (3) eosinophilic inflammation and *H. influenzae* colonisation rarely coexisted. These findings highlight the different COPD endotypes defined by sputum microbiology and inflammatory cell counts. 

## Figures and Tables

**Table 1:** Demographics for COPD subjects and healthy non-smoking controls. Summaries are presented as percentages, Mean (SD) or median [range] as appropriate (*n* = 60 and 15, respectively *).

Characteristic	COPD *n* = 60	Healthy Non-Smokers *n* = 15	*p*-Value
Gender (% Male)	58.3	60.0	0.91
Age	64.9 (7.3)	59.0 (10.4)	0.02
Smoking status (Current %)	43.3	0.0	n/a
Pack years	43.9 (18.9)	n/a	n/a
BMI (kg/m^2^)	28.4 (5.7)	26.2 (3.2)	0.12
Retrospective Exacerbation rate (1-year period)	1.1 (1.3)	n/a	n/a
0 (%)	41.6	n/a	n/a
1 (%)	31.7	n/a	n/a
≥2 (%)	26.7	n/a	n/a
Prospective annualised exacerbation rate (annualised to a 1-year period)	1.2 (1.8)	n/a	n/a
^a^ FEV_1_ (L)	1.8 (0.6)	3.1 (0.8)	<0.01
^a^ FEV_1_ (%)	66.7 (16.6)	105.5 (11.9)	<0.01
^a^ FEV_1_/FVC Ratio (%)	54.0 (11.3)	75.9 (4.7)	<0.01
GOLD Category (%)			
1	26.7	n/a	n/a
2	55.0	n/a	n/a
3	18.3	n/a	n/a
4	0	n/a	n/a
CAT	22.3 (5.6)	n/a	n/a
mMRC	4.0 [2.0–4.0]	n/a	n/a
SGRQ-C (Total)	54.2 (16.1)	n/a	n/a
Atopy (%)	12.1	20.0	0.42
Chronic bronchitis (%)	83.3	n/a	n/a
ICS Use (%)	71.7	n/a	n/a
LABA + LAMA + ICS (%)	58.3	n/a	n/a
LABA + LAMA (%)	0.0	n/a	n/a
ICS only (%)	1.7	n/a	n/a
LABA only (%)	0.0	n/a	n/a
LAMA only (%)	15.0	n/a	n/a
No inhaled medication (%)	5.0	n/a	n/a
Sputum characteristics			
Sputum total cell count × 10^6^/g	8.25 [0.62–100.9]	7.60 [2.81–20.48]	0.39
Sputum Neutrophil (%)	69.13 [24.25–97.75]	70.50 [37.50–88.50]	0.32
Sputum Eosinophil (%)	1.00 [0.00–16.50]	0.00 [0.00–4.25]	<0.01
Sputum Lymphocyte (%)	0.50 [0.00–4.75]	0.50 [0.00–3.00]	0.49
Sputum Macrophage (%)	21.00 [1.00–68.00]	27.00 [6.25–58.50]	0.17
Sputum Epithelial Cells (%)	1.63 [0.00–16.50]	2.75 [0.00–14.25]	0.35
Sputum Neutrophil cell count × 10^6^/g	5.22 [0.32–98.08]	5.04 [1.24–14.74]	0.33
Sputum Eosinophil cell count × 10^6^/g	0.08 [0.00–2.45]	0.00 [0.00–0.79]	<0.01
Sputum Lymphocyte cell count × 10^6^/g	0.03 [0.00–0.64]	0.04 [0.00–0.33]	0.92
Sputum Macrophage cell count × 10^6^/g	1.28 [0.20–7.57]	2.06 [0.38–5.53]	0.30
Sputum Epithelial cell count × 10^6^/g	0.16 [0.00–1.59]	0.17 [0.00–1.42]	0.78
Total PPM Load (genome copies/mL)	9.01 × 10^4^ [0.00–1.58 × 10^8^]	1.31 × 10^5^ [0.00–7.09 × 10^6^]	0.86
HI Load (genome copies/mL)	1.94 × 10^3^ [0.00–1.58 × 10^8^]	1.05 × 10^3^ [0.00–3.22 × 10^5^]	0.17
SP Load (genome copies/mL)	3.41 × 10^3^ [0.00–1.82 × 10^7^]	2.52 × 10^4^ [0.00–7.09 × 10^6^]	0.23
MC Load (genome copies/mL)	0.00 [0.00–9.22 × 10^6^]	0.00 [0.00–3.72 × 10^3^]	0.39
PA Load (genome copies/mL)	0.00 [0.00–5.88 × 10^6^]	0.00 [0.00–1.68 × 10^2^]	0.12

* The following data were missing for COPD subjects: 2 atopy status and due to insufficient sputum sample in the COPD patients, 7 sputum DCC and 18 bacterial qPCR data. ^a^ FEV_1_ (L and % predicted) are post-BD values for COPD and pre-BD values for HNS. BD, bronchodilator; BMI, body mass index; CAT, COPD assessment test; DCC, differential cell count; FEV_1_, forced expiratory volume in 1 s; FVC, forced vital capacity; HI, *Haemophilus influenzae*; ICS, inhaled corticosteroids; LABA, long acting beta agonist; LAMA, long acting muscarinic antagonist; MC, *Moraxella catarrhalis*; mMRC, modified medical research council questionnaire; PPM, potentially pathogenic microorganism; PA, *Pseudomonas aeruginosa*; SGRQ, St George’s respiratory questionnaire; SP, *Streptococcus pneumoniae*.

**Table 2:** Presence of PPMs as defined by HNS thresholds in COPD patients with sputum eosinophils persistently ≥3%, ≥3% at one visit only or <3% at both visits. *n* = 7, 8 and 33, respectively.

	Sputum Eos Persistently ≥3% (*n* = 7)		Sputum Eos ≥3% at One Visit Only (*n* = 8)		Sputum Eos <3% at Both Visits (*n* = 33)	
	Baseline	6 months	Baseline	6 months	Baseline	6 months
No PPM	6	6	6	6	12	11
HI	0	0	1	1	7	7
PA	0	0	0	0	1	0
SP	0	0	0	0	0	1
MC	0	1	0	1	0	1
^a^ >1 PPM	0	0	0	0	1	2
Any bacterial colonisation	0/6 (0.0%)	1/7 (14.3%)	1/7 (14.3%)	2/8 (25.0%)	9/21 (42.9%)	11/22 (50.0%)
* No data	1	0	1	0	12	11

* Data were unavailable due to insufficient sputum sample obtained. ^a^ >1 PPM group consisted of HI + MC at baseline and HI + MC or MC + PA at 6 months. Eos, eosinophil; HI, *Haemophilus influenzae*; MC, *Moraxella catarrhalis*; PPM, potentially pathogenic microorganism; PA, *Pseudomonas aeruginosa*; SP, *Streptococcus pneumoniae*; >1 PPM more than one potentially pathogenic microorganism.

## Data Availability

The datasets generated and/or analysed during the current study are not publicly available.

## Supplementary Materials

The following are available online at https://www.mdpi.com/article/10.3390/biomedicines9101337/s1, Figure S1. Flow diagram to show selection of COPD subjects. *n* = 60, Figure S2. Association between age and baseline *H. influenzae* and total bacterial load of COPD patients (a,b) and HNS (c,d). *n* = 52 and 15, respectively, Figure S3. Sputum neutrophil and eosinophil percentages for COPD patients colonised with different PPMs at baseline (*n* = 49) and 6-month (*n* = 38) visits. Sputum differential cell counts were performed on patients with no colonisation (none), colonisation with *Haemophilus influenzae* only, *Streptococcus pneumoniae* only, *Moraxella catarrhalis* only, *Pseudomonas aeruginosa* only or more than one PPM. Data represent sputum neutrophil or eosinophil percentages for individual patients; (a,b) at baseline, or (c,d) at 6 months, Table S1. Details of qPCR targets and the lower limits of detection for qPCR detection of different PPMs, Table S2. Concomitant disease for COPD patients and healthy controls, Table S3. Characteristics for ICS users vs. non-users, Table S4. Sputum characteristics for current smoking COPD patients vs. ex-smoking, for those with qPCR data only, Table S5. Baseline characteristics for HI^+ve^, HI^−ve^ patients and those with no PPMs, Table S6. The 6-month sputum characteristics for HI^+ve^, HI^−ve^ patients and those with no PPMs.
