# Supplementary material for: Airway Bacteria Quantification Using Polymerase Chain Reaction Combined with Neutrophil and Eosinophil Counts Identifies Distinct COPD Endotypes
Source: Biomedicines. 2021 Sep 27;9(10):1337. doi: 10.3390/biomedicines9101337 (PMC8533560; doi:10.3390/biomedicines9101337)
Supplement: Supplementary file 1 [file biomedicines-09-01337-s001.zip › biomedicines-1375942-supplementary.pdf]

Supplementary Material

Airway bacteria quantification using polymerase chain reaction combined with neutrophil and eosinophil counts identifies distinct COPD endotypes

Augusta Beech, Simon Lea, Jian Li, Natalie Jackson, Alex Mulvanny and Dave Singh

Methods

Sputum measurements

Prior to processing, selected sputum plugs were weighed – samples with a weight <0.1g were not processed due to insufficient sample size. Each sputum sample was objectively assessed for quality; if the leukocyte viability was <50% and/or the squamous cell percentage was >30%, the sample was discarded on the basis of poor quality.

qPCR detection of common respiratory pathogens

DNA was extracted from sputum samples using QIAamp DNA mini kit (QIAGEN). Real-time qPCR was performed as previously described [1, 2]. The qPCR program consisted of 95 °C for 15 minutes, 40 cycles of 95 °C for 15 seconds and 60 °C for 1 minute with a plate read after every cycle. Fluorescence was detected and captured using an Agilent MX3005P detection system. Automated analysis using ABI. Prism software calculated the Ct values, Prism version 9.00 (San Diego, USA). A standard curve of known concentrations of bacteria was used to calculate bacteria level.

Results

Bacterial Colonisation

The clinical characteristics of HI^{+ve} and HI^{-ve} patients were compared. The HI^{+ve} group was comprised of all patients with evidence of *H.influenzae* colonisation, regardless of co-colonisation; 1 patient (8.3%) was colonised with *H.influenzae* and *M.catarrhalis* and 1 patient (8.3%) was colonised with *H.influenzae*, *S.pneumoniae* and *M.catarrhalis.* The HI^{-ve} group was comprised of all patients without evidence of *H.influenzae* colonisation, this group included n=1 (2.5%) patient colonised with *P.aeruginosa* (n=40). There was a significantly lower proportion of male participants in the HI^{-ve} group, clinical characteristics were otherwise similar between groups. When the clinical characteristics of HI^{+ve} and HI^{-ve}

patients were compared excluding the n=1 patient colonised with *P.aeruginosa* (No PPM group), results were similar (n=39, Table S5).

Relationship between colonisation and sputum cell counts

At baseline, HI^{+ve} patients had a lower macrophage percentage compared to HI^{-ve} patients; (7.1 and 25.3% respectively, p<0.01, Table S5) and a higher total sputum cell count (16.2 and $7.5x10^6$ cells/g, p=0.01). When the baseline clinical characteristics of HI^{+ve} and HI^{-ve} patients were compared excluding the n=1 patient colonised with *P.aeruginosa* (No PPMs), results were similar (n=39, Table S5). When comparing HI^{+ve} patients to those with no PPMs at baseline, results were similar to comparisons of HI^{+ve} versus HI^{-ve} patients (Table S5).

Data from 6 month visits showed similar results to baseline; HI^{+ve} patients had a lower macrophage percentage compared to HI^{-ve} patients; (12.4 and 23.6% respectively, p=0.02, Table S6) and a higher total sputum cell count (11.6 and $6.9x10^6$ cells/g, p=0.03). When comparing HI^{+ve} patients to those with no PPMs, results were similar aside from a significantly lower sputum epithelial percentage in HI^{+ve} patients (3.0 and 1.1% for those with no PPM and HI^{+ve} respectively, p=0.04, Table S6).

Using the upper threshold of the HNS range to subgroup COPD patients based on qPCR detection of potentially pathogenic microorganisms, sputum neutrophil and eosinophil counts were directly compared between groups. At baseline sputum neutrophil count was significantly higher in the HI group compared to those with no PPM colonisation (89.8 versus 66.6%, p=0.04, Figure S3a) whilst the sputum eosinophil count was numerically lower in this group (0.13 and 0.5 versus 2.0%, Figure S3b), however the difference was not statistically significant. No other differences were observed between groups at baseline. At 6 months sputum neutrophil counts were significantly higher in both HI

and >1PPM groups compared to those with no PPM colonisation (86.0 and 86.0 versus 68.8%, $p=0.02$ and 0.04 respectively, Figure S3c) whilst the sputum eosinophil count was lower in the HI group (0.13 versus 2.0%, $p<0.01$, Figure S3d).

Figure legends

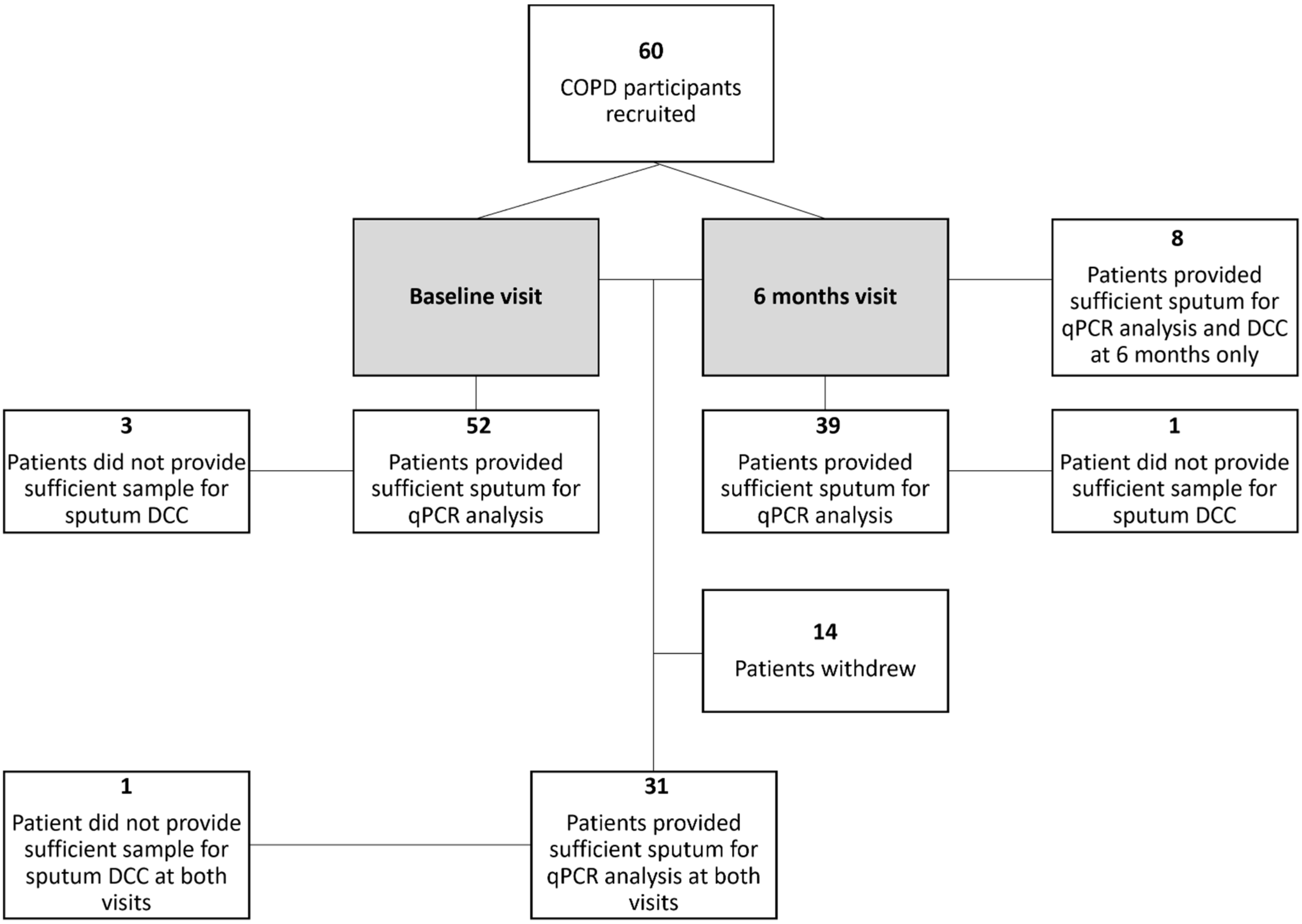

Figure S1: Flow diagram to show selection of COPD subjects. n=60.

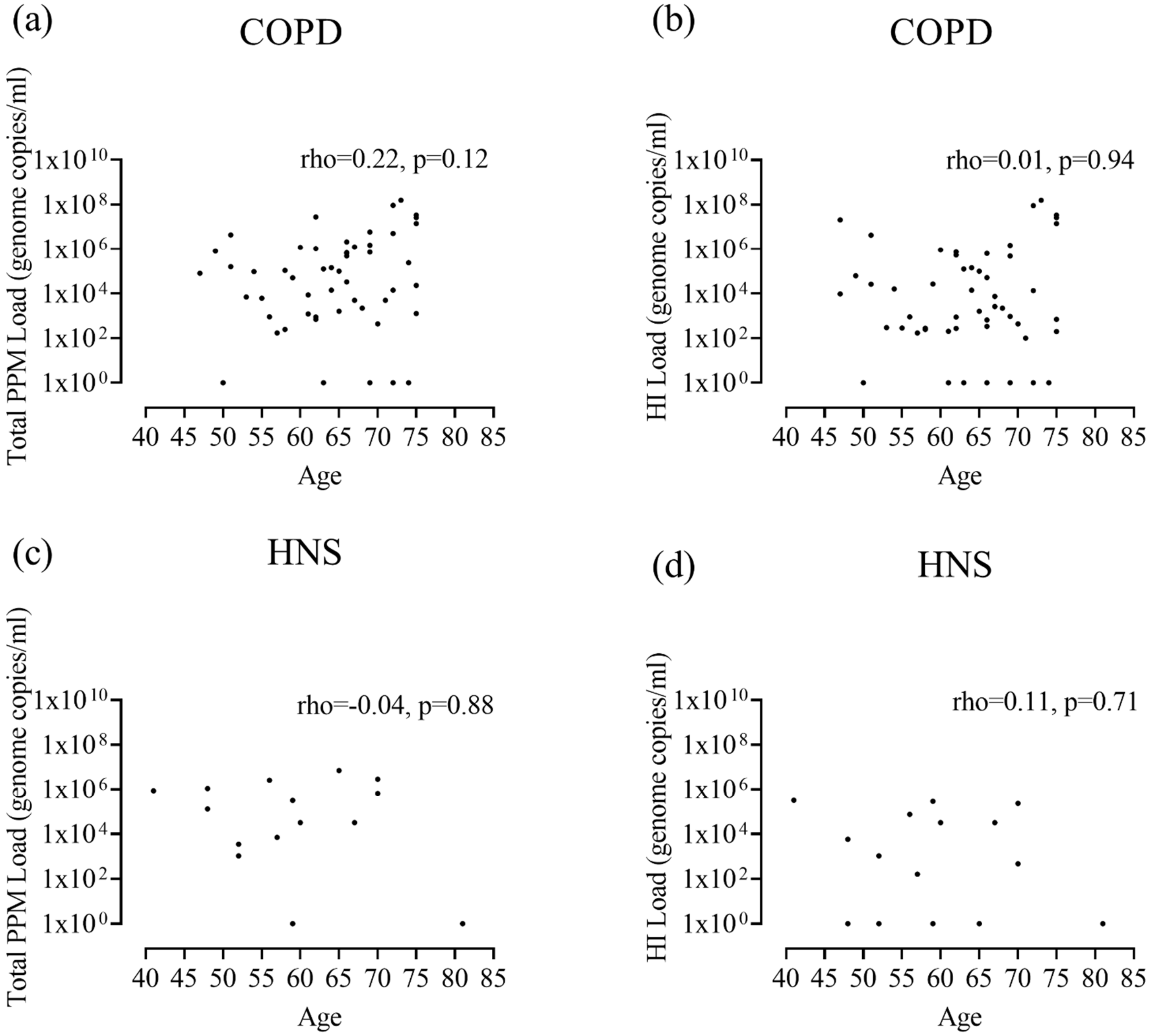

Figure S2: Association between age and baseline *H.influenzae* and total bacterial load of COPD patients (a & b) and HNS (c & d). n= 52 and 15 respectively. Chronic obstructive pulmonary disease, COPD; *Haemophilus influenzae*, HI; potentially pathogenic microorganism, PPM.

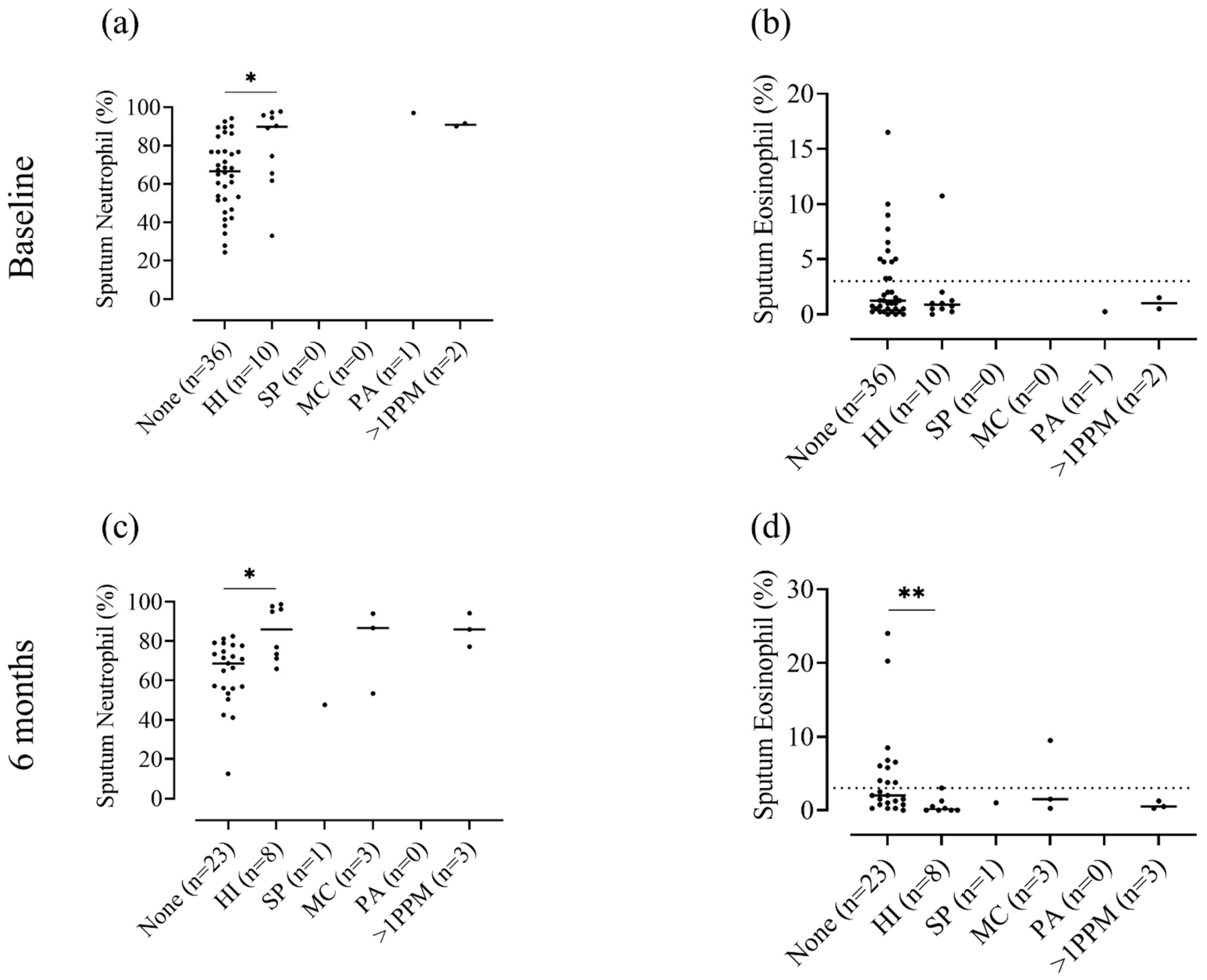

Figure S3: Sputum neutrophil and eosinophil percentages for COPD patients colonised with different PPMs at baseline (n=49) and 6 month (n=38) visits. Sputum differential cell counts were performed on patients with no colonisation (none), colonisation with *Haemophilus influenzae* only, *Streptococcus pneumoniae* only, *Moraxella catarrhalis* only, *Pseudomonas aeruginosa* only or more than one PPM. Data represents sputum neutrophil or eosinophil percentages for individual patients; (a & b) at baseline, or (c & d) at 6 months. Chronic obstructive pulmonary disease, COPD; *Haemophilus influenzae*, HI; *Moraxella catarrhalis*, MC; no colonisation, None; potentially pathogenic microorganism, PPM; *Pseudomonas aeruginosa*, PA; *Streptococcus pneumoniae*, SP.

__Tables__

Table S1: Details of qPCR targets and the lower limits of detection for qPCR detection of different PPMs.

Target species	Gene target	Primers	Sequence (5′-3′)	Probe sequence with Reporter and Quencher	Lower limit of detection (genome copies/ml)
HI	P4 Lipoprotein gene	HI-F	CCgggTgCggTAgAATTTAATAA	6FAM-ACAGCCACAACGGTAAAGTGTTCTACG-DB	8.70x10^{1}
		HI-R	CTgATTTTTCAgTgCTgTCTTTgC		
SP	Spn9082 gene fragment	SP-F	AgTCgTTCCAAggTAACAAgTCT	ROX-TACATGTAGGAAACTATTTTCCTCACAAA-BHQ2	1.38x10^{3}
		SP-R	ACCAACTCgACCACCTCTTT		
MC	CopB outer membrane protein gene	MC-F	gTgAgTgCCgCCAAgACAA	6JOE-TGCTTTTGCAGCTGTTAGCCAGCCTAA-BHQ1	1.76x10^{2}
		MC-R	TgTATCgCCTgCCAAgACAA		
PA	gyrB gene	PA-F	TCCAAGTTTAAGGTGGTAGGCTG	FAM-AGG TAA ATC CGG GGT TTC AAG GCC-TAMRA	6.00
		PA-R	ACCACTTCGTCAATCTAAAAGACGA		

F, Forward; HI, *Haemophilus influenzae*; MC, *Moraxella catarrhalis*; PA, *Pseudomonas aeruginosa*; qPCR, quantitative polymerase chain reaction; R, reverse; SP, *Streptococcus pneumoniae*.

Table S2: Concomitant disease for COPD patients and healthy controls.

Concomitant disease	COPD (n=60) n (%)	HNS (n=15) n (%)	p-value
Patients with at least one concomitant disease	57 (95.0)	6 (40.0)	<0.01
Ischemic heart disease	16 (26.7)	0 (0.0)	0.02
Myocardial ischemia	10 (16.7)	0 (0.0)	0.09
Angina pectoris	6 (10.0)	0 (0.0)	0.20
Myocardial infarction	9 (15.0)	0 (0.0)	0.11
Cardiac failure	0 (0.0)	0 (0.0)	n/a
Cardiovascular disease	45 (75.0)	3 (20.0)	<0.01
Hypertension	30 (50.0)	3 (20.0)	0.04
Hypercholesterolemia	35 (58.3)	3 (20.0)	<0.01
Coronary artery disease	5 (8.3)	0 (0.0)	0.25
Pulmonary hypertension	0 (0.0)	0 (0.0)	n/a
Peripheral vascular disease	2 (3.3)	0 (0.0)	0.47
Cerebrovascular disease	6 (10.0)	0 (0.0)	0.20
Stroke (including transient ischaemic attack)	6 (10.0)	1 (6.7)	0.69
Irregular heartbeat	2 (3.3)	0 (0.0)	0.47
Diabetes	8 (13.3)	0 (0.0)	0.13
Obesity	24 (40.0)	2 (13.3)	0.05
Obstructive sleep apnoea	1 (1.7)	0 (0.0)	0.61
Anaemia	4 (6.7)	3 (20.0)	0.11
Osteoarthritis	19 (31.7)	0 (0.0)	0.01
Osteoporosis	2 (3.3)	1 (6.7)	0.56
Gastro-oesophageal reflux disease	15 (25.0)	1 (6.7)	0.12
Psychological disturbances	17 (28.3)	2 (13.3)	0.23
Depression	16 (26.7)	2 (13.3)	0.28
Anxiety	3 (5.0)	0 (0.0)	0.38
Insomnia	1 (1.7)	0 (0.0)	0.61

Summaries are presented as n (%), n=60 and 15 respectively.

Table S3: Characteristics for ICS users versus non-users.

Characteristic	ICS Users n=43	Non users n=17	p-value
Gender (% Male)	60.5	52.9	0.59
Age	65.4 (7.3)	63.8 (7.4)	0.44
Smoking status (Current %)	39.5	52.9	0.35
Pack years	42.6 (19.1)	47.2 (18.5)	0.40
BMI (kg/m^2)	28.5 (5.6)	28.1 (6.0)	0.78
Exacerbations (1 year period)	1.3 (1.4)	0.6 (0.8)	0.07
0 (%)	34.9	58.9	0.09
1 (%)	34.9	23.5	0.39
≥2 (%)	30.2	17.6	0.32
Post FEV1 (L)	1.8 (0.6)	1.9 (0.6)	0.53
Post FEV1 (%)	65.4 [30.2-96.6]	67.8 [42.3-90.0]	0.79
GOLD Category (%)			
1	27.9	23.5	0.73
2	51.2	64.7	0.34
3	20.9	11.8	0.41
4	0.0	0.0	n/a
CAT	21.0 [15.0-32.0]	20.0 [15.0-39.0]	0.68
mMRC	4.0 [2.0-4.0]	2.0 [2.0-4.0]	0.71
SGRQ-C (Total)	54.7 (16.7)	53.0 (19.6)	0.72
Atopy (%)	11.9	12.5	>0.99
Chronic bronchitis (%)	88.4	70.6	0.10
**Sputum characteristics**			
Sputum total cell count x106/g	8.80 [0.62-100.9]	7.11 [1.43-35.15]	0.70
Sputum Neutrophil (%)	70.63 [33.0-97.75]	67.13 [24.25-95.75]	0.47
Sputum Eosinophil (%)	1.25 [0.00-16.50]	0.88 [0.00-5.00]	0.15
Sputum Lymphocyte (%)	0.38 [0.00-4.75]	0.50 [0.00-2.75]	0.34
Sputum Macrophage (%)	17.25 [1.00-56.25]	27.25 [2.00-68.00]	0.25
Sputum Epithelial Cells (%)	1.88 [0.00-16.50]	1.50 [0.25-16.25]	0.78
Sputum Neutrophil cell count x10^6/g	5.56 [0.32-98.08]	3.99 [0.35-33.66]	0.65
Sputum Eosinophil cell count x10^6/g	0.11 [0.00-2.45]	0.04 [0.00-0.36]	0.13
Sputum Lymphocyte cell count x10^6/g	0.03 [0.00-0.64]	0.04 [0.00-0.26]	0.39
Sputum Macrophage cell count x10^6/g	1.34 [0.20-7.57]	1.26 [0.59-3.50]	0.63
Sputum Epithelial cell count x10^6/g	0.13 [0.00-1.59]	0.22 [0.02-0.57]	0.72
Total PPM Load (genome copies/ml)	9.01E+04 [0.00E+00-9.25E+07]	1.77E+05 [0.00E+00-1.58E+08]	0.69
HI Load (genome copies/ml)	2.15E+03 [0.00E+00-9.05E+07]	1.09E+04 [0.00E+00-1.58E+08]	0.85
SP Load (genome copies/ml)	1.56E+03 [0.00E+00-1.82E+07]	2.86E+04 [0.00E+00-1.25E+06]	0.61

MC Load (genome copies/ml)	0.00E+00 [0.00E+00-9.22E+06]	0.00E+00 [0.00E+00-9.42E+02]	0.61
PA Load (genome copies/ml)	0.00E+00 [0.00E+00-5.88E+06]	0.00E+00 [0.00E+00-0.00E+00]	>0.99

Summaries are presented as percentages, mean (SD) or median [range] as appropriate, n=43 and 17, respectively.

The following data were not available for COPD; 2 atopy status and due to insufficient sputum sample; 4 DCC and cell counts, 18 qPCR.

BD, bronchodilator; BMI, body mass index; CAT, COPD assessment test; differential cell count, DCC; FEV_1, forced expiratory volume in 1 second; FVC, forced vital capacity; *Haemophilus influenzae*, HI; ICS, inhaled corticosteroids; LABA, long acting beta agonist; LAMA, long acting muscarinic antagonist; *Moraxella catarrhalis*, MC; mMRC, modified medical research council questionnaire; PPM, potentially pathogenic microorganism; PA, *Pseudomonas aeruginosa*; SGRQ, St George's respiratory questionnaire; and SP, *Streptococcus pneumoniae*.

Table S4: Sputum characteristics for current smoking COPD patients versus ex-smoking, for those with qPCR data only.

**Sputum characteristics of COPD patients with bacterial qPCR data**	**Current smoker n=24**	**Ex-smoker n=28**	**p-value**
Total PPM Load (genome copies/ml)	7.43E+04 [0.00E+00-4.38E+06]	1.63E+05 [0.00E+00-1.58E+08]	0.14
HI Load (genome copies/ml)	9.03E+02 [0.00E+00-4.27E+06]	2.45E+03 [0.00E+00-1.58E+08]	0.81
SP Load (genome copies/ml)	4.65E+03 [0.00E+00-7.48E+05]	1.23E+03 [0.00+00-1.82+07]	0.96
MC Load (genome copies/ml)	0.00E+00 [0.00E+00-9.42E+02]	0.00E+00 [0.00E+00-9.22E+06]	0.45
PA Load (genome copies/ml)	0.00E+00 [0.00E+00-0.00E+00]	0.00E+00 [0.00E+00-5.88E+06]	>0.99

Summaries are presented as median [range], n=24 and 28 respectively. HI, *Haemophilus influenzae*; MC, *Moraxella catarrhalis*; PPM, potentially pathogenic microorganism; PA, *Pseudomonas aeruginosa*; SP, *Streptococcus pneumoniae*

Table S5: Baseline characteristics for HI^{+ve}, HI^{-ve} patients and those with no PPMs.

Characteristic	HI^{+ve} n=12	HI^{-ve} n=40	No PPMs n=39
Gender (% Male)	83.3	***47.5**	***48.7**
Age	67.4 (7.5)	63.4 (7.6)	63.3 (7.6)
Smoking status (Current %)	25.0	52.5	53.8
Pack years	51.7 (18.0)	43.1 (18.6)	43.1 (18.6)
BMI (kg/m^2)	27.8 (5.2)	28.4 (5.9)	28.4 (6.0)
Retrospective exacerbation rate (1 year period)	1.3 (1.6)	1.0 (1.2)	1.0 (1.2)
0 (%)	41.7	45.0	46.2
1 (%)	25.0	32.5	33.3
≥2 (%)	33.3	22.5	20.5
Prospective exacerbation rate (annualised to 1 year period)	1.3 (1.7)	0.86 (1.4)	0.8 (1.3)
Post FEV1 (L)	1.8 (0.6)	1.8 (0.5)	1.8 (0.5)
Post FEV1 (%)	61.6 (15.4)	68.2 (15.0)	68.1 (15.2)
GOLD Category (%)			
1	25.0	15.0	15.4
2	58.3	57.5	56.4
3	16.7	27.5	28.2
4	0.0	0.0	0.0
CAT	22.0 [15.0-39.0]	21.0 [15.0-31.0]	21.0 [15.0-31.0]
mMRC	4.0 [2.0-4.0]	4.0 [2.0-4.0]	4.0 [2.0-4.0]
SGRQ-C (Total)	58.0 (15.9)	53.2 (15.4)	52.9 (15.5)
Atopy (%)	16.7	8.1	5.4
Chronic bronchitis (%)	83.3	85.0	84.6
ICS Use %	66.7	70.0	69.2
**Sputum characteristics**			
Sputum total cell count x10^6/g	16.23 [4.44-100.9]	***7.49 [0.62-42.61]**	****7.33 [0.62-42.61**
Sputum Neutrophil (%)	90.13 [33.0-97.75]	****67.25 [24.25-97.00]**	****66.63 [24.25-94.25]**
Sputum Eosinophil (%)	0.88 [0.00-10.75]	1.25 [0.00-16.50]	1.25 [0.00-16.50]
Sputum Lymphocyte (%)	0.25 [0.00-1.00]	0.50 [0.00-4.75]	0.50 [0.00-4.75]
Sputum Macrophage (%)	7.13 [1.00-41.25]	****25.25 [1.50-68.00]**	****25.88 [3.75-68.00]**
Sputum Epithelial Cells (%)	1.00 [0.00-14.50]	2.00 [0.0-16.50]	2.13 [0.00-16.50]
Sputum Neutrophil cell count x10^6/g	14.65 [2.91-98.08]	***4.65 [0.32-36.11]**	***4.61 [0.32-36.11]**
Sputum Eosinophil cell count x10^6/g	0.10 [0.00-1.18]	0.10 [0.00-2.45]	0.10 [0.00-2.45]

Sputum Lymphocyte cell count x10⁶/g	0.06 [0.00-0.63]	0.03 [0.00-0.64]	0.03 [0.00-0.64]
Sputum Macrophage cell count x10⁶/g	1.13 [0.25-4.53]	1.29 [0.20-7.57]	1.35 [0.20-7.57]
Sputum Epithelial cell count x10⁶/g	0.20 [0.00-1.59]	0.13 [0.00-1.29]	0.12 [0.00-1.29]
Total PPM Load (genome copies/ml)	9.34E+06 [6.48E+05-1.58E+08]	****1.15E+04 [0.00E+00-5.88E+06]**	****8.73E+03 [0.00E+00-5.12E+06]**
HI Load (genome copies/ml)	2.88E+06 [4.89E+05-1.58E+08]	****5.49E+02 [0.00E+00-1.44E+05]**	****4.42E+02 [0.00E+00-1.44E+05]**
SP Load (genome copies/ml)	8.14E+04 [0.00E+00-1.82E+07]	0.00E+00 [0.00E+00-5.12+06]	0.00E+00 [0.00E+00-5.12E+06]
MC Load (genome copies/ml)	0.00E+00 [0.00E+00-9.22E+06]	****0.00E+00 [0.00E+00-6.59E+02]**	****0.00E+00 [0.00E+00-6.59E+02]**
PA Load (genome copies/ml)	0.00E+00 [0.00E+00-0.00E+00]	0.00E+00 [0.00E+00-5.88E+06]	0.00E+00 [0.00E+00-0.00E+00]

The HI^{+ve} group was comprised of all patients with evidence of *H.influenzae* colonisation, regardless of co-colonisation including 1 patient colonised with *H.influenzae* and *M.catarrhalis* and 1 patient with *H.influenzae*, *S.pneumoniae* and *M.catarrhalis.* The HI^{-ve} group was comprised of all patients without evidence of *H.influenzae* colonisation, this group included n=1 patient colonised with *P.aeruginosa* (n=40).

Summaries are presented as percentages, Mean (SD) or median [range] as appropriate. n=12, 40 and 39, respectively.

* p<0.05 when compared to HI^{+ve} patients

** p<0.05 when compared to HI^{+ve} patients

The following data were not available; 2 atopy status, and due to insufficient sputum sample; 3 DCC and cell counts.

BD, bronchodilator; BMI, body mass index; CAT, COPD assessment test; differential cell count, DCC; FEV1, forced expiratory volume in 1 second; FVC, forced vital capacity; HI, *Haemophilus influenzae*; ICS, inhaled corticosteroids; LABA, long acting beta agonist; LAMA, long acting muscarinic antagonist; MC, *Moraxella catarrhalis*; mMRC, modified medical research council questionnaire; PA, *Pseudomonas aeruginosa*; PPM, potentially pathogenic microorganism; SGRQ, St George's respiratory questionnaire; and SP, *Streptococcus pneumoniae*.

Table S6: 6 month sputum characteristics for HI^{+ve}, HI^{-ve} patients and those with no PPMs.

Sputum characteristics	HI^{+ve} n=10	HI^{-ve} n=29	No PPMs n=24
Sputum total cell count x10^6/g	11.63 [6.57-38.65]	***6.87 [1.85-30.53]**	***6.89 [1.85-24.38]**
Sputum Neutrophil (%)	81.63 [66.0-98.75]	****69.88 [12.50-94.25]**	****68.75 [12.50-82.50]**
Sputum Eosinophil (%)	0.25 [0.00-3.00]	****1.75 [0.00-24.00]**	****2.00 [0.00-24.00]**
Sputum Lymphocyte (%)	0.50 [0.00-1.500]	0.25 [0.00-2.00]	0.25 [0.00-2.00]
Sputum Macrophage (%)	12.38 [1.00-30.00]	***23.63 [2.00-57.25]**	***24.00 [9.75-57.25]**
Sputum Epithelial Cells (%)	1.13 [0.00-6.25]	2.38 [0.00-30.00]	***3.00 [0.00-30.00]**
Sputum Neutrophil cell count x10^6/g	8.52 [4.33-36.72]	***4.73 [0.23-28.70]**	****4.79 [0.23-19.01]**
Sputum Eosinophil cell count x10^6/g	0.03 [0.00-1.16]	0.11 [0.00-2.25]	0.13 [0.00-2.25]
Sputum Lymphocyte cell count x10^6/g	0.06 [0.00-0.17]	0.01 [0.00-0.27]	0.01 [0.00-0.27]
Sputum Macrophage cell count x10^6/g	1.05 [0.25-3.31]	1.64 [0.11-3.97]	1.59 [0.44-3.97]
Sputum Epithelial cell count x10^6/g	0.22 [0.00-0.58]	0.16 [0.00-0.82]	0.19 [0.00-0.82]
Total PPM Load (genome copies/ml)	1.37E+07 [2.17E+06-2.05E+08]	****4.30E+05 [1.27E+01-2.59E+08]**	****1.25E+05 [1.27E+01-5.60E+06]**
HI Load (genome copies/ml)	1.29E+07 [2.15E+05-2.05E+08]	****3.07E+03 [0.00E+00-1.41+05]**	****3.10E+03 [0.00+00-1.31E+05]**
SP Load (genome copies/ml)	4.35E+05 [0.00E+00-1.01E+07]	1.15E+05 [0.00E+00-2.58E+07]	1.04E+05 [0.00E+00-5.59E+06]
MC Load (genome copies/ml)	0.00E+00 [0.00E+00-1.63E+06]	**0.00E+00 [0.00E+00-1.25E+07]**	***0.00E+00 [0.00E+00-1.18E+03]**
PA Load (genome copies/ml)	0.00E+00 [0.00E+00-0.00E+00]	0.00E+00 [0.00E+00-2.39E+06]	0.00E+00 [0.00E+00-0.00E+00]

The HI^{+ve} group was comprised of all patients with evidence of *H.influenzae* colonisation, regardless of co-colonisation including 1 patient colonised with *H.influenzae* and *M.catarrhalis* and 1 patient with *H.influenzae, S.pneumoniae* and *M.catarrhalis.* The HI^{-ve} group was comprised of all patients without evidence of *H.influenzae* colonisation, this group included n=1 patient colonised with *P.aeruginosa* (n=40).

Summaries are presented as percentages, Mean (SD) or median [range] as appropriate, n=10, 29 and 24 respectively.

The following data were not available due to insufficient sputum sample; 1 DCC and cell counts.

* p<0.05 when compared to HI^{+ve} patients

** p<0.05 when compared to HI^{+ve} patients

DCC, Differential cell count; HI *Haemophilus influenzae*; MC *Moraxella catarrhalis*; PPM, potentially pathogenic microorganism; PA, *Pseudomonas aeruginosa*; SP, *Streptococcus pneumoniae*.

References

1. Garcha, D.S., et al., *Changes in prevalence and load of airway bacteria using quantitative PCR in stable and exacerbated COPD.* Thorax, 2012. **67**(12): p. 1075-80.
2. McCulloch, E., et al., *Improved early diagnosis of Pseudomonas aeruginosa by real-time PCR to prevent chronic colonisation in a paediatric cystic fibrosis population.* J Cyst Fibros, 2011. **10**(1): p. 21-4.
